# Detection of gene clusters for biodegradation of alkanes and aromatic compounds in the Rhodococcus qingshengii VKM Ac-2784D genome

**DOI:** 10.18699/VJGB-23-33

**Published:** 2023-06

**Authors:** Yu.A. Markova, I.S. Petrushin, L.A. Belovezhets

**Affiliations:** Siberian Institute of Plant Physiology and Biochemistry of the Siberian Branch of the Russian Academy of Sciences, Irkutsk, Russia; Siberian Institute of Plant Physiology and Biochemistry of the Siberian Branch of the Russian Academy of Sciences, Irkutsk, Russia Irkutsk State University, Irkutsk, Russia; A.E. Favorsky Irkutsk Institute of Chemistry of the Siberian Branch of the Russian Academy of Sciences, Irkutsk, Russia

**Keywords:** biodegradation, Rhodococcus, oil destruction, genomics, биодеструкция, Rhodococcus, нефтедеструкция, геномика

## Abstract

Bacterial species of the genus Rhodococcus are known to be efficient degraders of hydrocarbons in contaminated soil. They are also employed for bioremediation of polluted environments. These bacteria are widely met in soil, water and living organisms. Previously, we have isolated the Rhodococcus qingshengii strain VKM Ac-2784D from the rhizosphere of couch grass growing on oil-contaminated soil. This strain can effectively degrade oil and some model compounds (naphthalene, anthracene and phenanthrene). The results of phylogenetic analysis show that this strain belongs to the species R. qingshengii. To understand the catabolic properties of this strain, we have studied its gene clusters possessing such properties. The alkane destruction genes are represented by two clusters and five separate alkB genes. The destruction of aromatic compounds involves two stages, namely central and peripheral. The R. qingshengii VKM Ac-2784D genome contains four out of eight known central metabolic pathways for the destruction of aromatic compounds. The structure of the gene clusters is similar to that of the known strains R. jostii RHA1 and R. ruber Chol-4. The peripheral pathways include the genes encoding proteins for benzoic acid destruction. The presence of biphenyl 2,3-dioxygeneses as well as gene clusters of benzoate and 2-hydroxypentandienoate pathways suggests that R. qingshengii VKM Ac-2784D could degrade polychlorinated biphenyls. The biodegradation ability can be enhanced by biosurfactants, which are known to be synthesized by Rhodococcus. The R. qingshengii VKM Ac-2784D genome contains the otsA, otsB, treY, treZ genes. The bioinformatics data are supported by the previous biochemical experiments that allow a mixture of species with a wide variation of metabolic pathways to be obtained.

## Abstract (ru)

Многие представители рода Rhodococcus известны как активные биодеструкторы компонентов нефти (в том числе алканов) и ароматических соединений. Пристальное внимание, которое стали уделять родококкам в последнее время, является следствием их высокого катаболического потенциала. Ранее нами выделен штамм Rhodococcus qingshengii VKM Ac-2784D из ризосферы пырея, произраставшего на нефтезагрязненной почве. По результатам филогенетического анализа этот штамм может быть отнесен к виду Rhodococcus qingshengii. На сегодняшний день расшифрованы пути и идентифицированы гены деструкции многих загрязнителей. Для оценки способности рассматриваемого штамма к деградации нефти и нефтепродуктов мы исследовали генные кластеры, ассоциированные с такой способностью. Ферменты деструкции алканов представлены двумя кластерами и пятью отдельно расположенными генами alkB. Деструкция ароматических соединений состоит из двух этапов: периферического и центрального. Геном R. qingshengii VKM Ac-2784D содержит четыре из восьми известных центральных путей деструкции ароматических соединений. Структура генных кластеров сходна с описанными в литературе штаммами R. jostii RHA1 и R. ruber Chol-4. Периферические пути представле- ны кластерами генов, кодирующих белки деструкции бензойной кислоты, ген бифенил 2,3-диоксигеназы и два гена бифенил-2,3-диол 1,2-диоксигеназы, участвующих в катаболизме бифенила. Присутствие генов бифенил 2,3-диоксигеназы, кластера генов бензоатного и 2-гидроксипентадиеноатного путей указывает на способность исследуемого штамма деструктировать полихлорированные бифенилы. Усилить активность биодеструкции загрязнителей помогают сурфактанты, улучшающие доступность разлагаемых веществ. Для некоторых видов Rhodococcus описаны биосурфактанты на основе трегалозы. В геноме R. qingshengii VKM Ac-2784D присутствуют гены, кодирующие белки биосинтеза сурфактантов – otsA, otsB, treY, treZ. Данные биоинформационного ана- лиза согласуются с результатами ранее проведенных биохимических исследований. Знание метаболического потенциала отдельного организма, полученное в результате анализа генома, позволит создавать адаптированные к заданным условиям смеси бактериальных штаммов, объединяющие микроорганизмы с разным спек- тром метаболических путей деструкции

## Introduction

Rhodococcus is a genus of Gram-positive bacteria of the Actinobacteria phylum, which are widespread in nature. These bacteria have been isolated from soil, water and living organisms. Some species of Rhodococcus are known to be pathogens, e. g. R. hoagii (former R. equi), which causes zoonotic infection, and R. fascians phytopathogen (Garrido- Sanz et al., 2020).

Over the last decade, rhodococci have attracted considerable interest owing to their high catabolic properties. These bacteria are able to degrade various pollutants (polyaromatic hydrocarbons, PAHs; dioxines, dioxin-like polychlorinated biphenyls, etc.) and, therefore, can be employed for bioremediation of soils (Martínková et al., 2009).

The application of rhodococci in bioactive mixtures requires an understanding of which pollutants are capable of destroying a microorganism. This goal can be achieved by the joint use of bioinformatic and biochemical approaches. Currently, most of the genes and pathways of pollutants degradation are known.

To date, numerous gene clusters of rhodococci genomes, which encode oil-degrading enzymes, have been reported (Zampolli et al., 2019). The key components of alkane degradation are alkane-monooxigenase (gene alkB, soluble di-iron monooxygenase, SDIMO) and cytochrome (CYP153, member of superfamily P450). Enzymes AlkB and CYP153 are usually involved in the oxidation of liquid alkanes, while SDIMO exerts an action on short-chain alkanes (<C8) (Coleman et al., 2011)

The destruction of aromatic compounds involves central and peripheral pathways. The latter are diverse: more than twenty such pathways are known in the literature. Owing to these pathways, rhodococci can oxidize polyaromatic hydrocarbons (PAHs), biphenyls, steroids or phthalates to common intermediates, which are further oxidized by central destruction routes. Eight central destruction pathways are described for Rhodococcus species: β-ketoadipate, phenylacetate, 2-hydroxypentadienoate, gentisate, homogentisate, hydroxyquinol, homoprotocatechuate, and a pathway with an unknown substrate (Guevara et al., 2019). Genes encoding proteins of peripheral pathways are usually located in plasmids, while genes of central pathways are localized in a chromosome.

Biosurfactants play an auxiliary role in the degradation of hydrophobic compounds. These surface-active agents (SAAs) improve bioavailability of the degrading substances. Rhodococci can synthesize the biosurfactants from trehalose and mycolic acids (Kuyukina, Ivshina, 2010).

In the present work, we tried to determine the genes encoding the above metabolic pathways in the Rhodococcus qingshengii VKM Ac-2784D genome as well as to evaluate the ability of this strain to degradation of oil components

## Materials and methods

The Rhodococcus qingshengii VKM Ac-2784D strain was isolated from the rhizosphere of couch grass (Elytrigia repens) growing on oil-contaminated soil near Tyret village, Irkutsk region, Russia (Belovezhets et al., 2017). It was found that this strain can oxidize both polyaromatic hydrocarbons (PAHs) and alkanes (Belovezhets et al., 2017).

To study genomic features and biodegradation potential, the whole genome was sequenced, assembled, annotated and submitted to NCBI GenBank (accession no. CP064920, Petrushin et al., 2021). The annotated R. qingshengii VKM Ac- 2784D genome contained 5775 genes encoding 5716 protein-coding sequences, 53 tRNAs, 3 noncoding RNAs (ncRNAs), and 3 rRNAs.

The sequence of the 16S rRNA subunit was assembled from the initial sequencing data using MATAM software (Pericard et al., 2018). To compare genomic features and define phylogenetic relationship of the R. qingshengii VKM Ac-2784D strain, the phylogenetic tree was built using 16S rRNA sequences of strains, described in recent reviews of Rhodococcus systematics (Gűrtler, Seviour, 2010; Sangal et al., 2019). The phylogenetic tree was built with MEGA X using the Tamura–Nei model and other settings set to default (Kumar et al., 2018). For close related species, average nucleotide identity distance matrix and phylogenetic tree were built using pyani v. 0.2.11 software, mode “ANIm” (https://github.com/ widdowquinn/pyani) with default settings. These distances were calculated by the MUMmer algorithm (Deloger et al., 2009) using whole genomes divided to fragments as described in (Richter, Rosselló-Móra, 2009).

Genes related to the metabolism of alkanes, biphenyls and other pollutants were located using BLAST search against whole genome of the R. qingshengii VKM Ac-2784D strain with known sequences of functional genes, described in previous studies. All figures, gene annotations were performed using UGENE software (Okonechnikov et al., 2012). Metabolic pathway models were analyzed in RAST SEED (Overbeek et al., 2014) online service.

## Results

Phylogenetic relationship of R. qingshengii VKM Ac-2784D

**Fig. 1.**
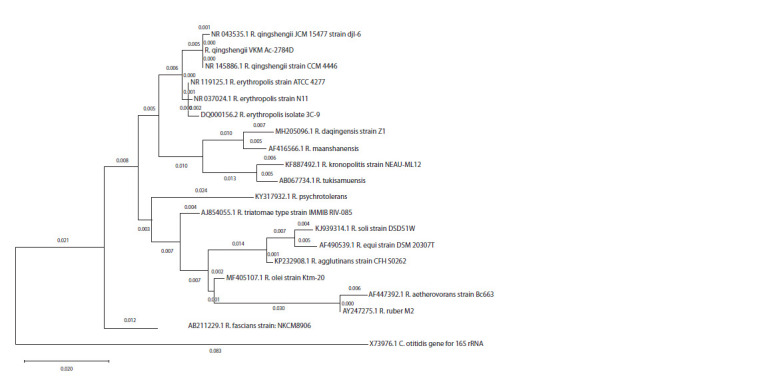
Phylogenetic tree of R. qingshengii VKM Ac-2784D and closer species The tree was constructed using 16S rRNA sequences of strains. Accession numbers in NCBI GenBank are given for each strain with its species name.

To identify phylogenetic relationship of the R. qingshengii VKM Ac-2784D strain, a traditional approach based on the construction of a phylogenetic tree according to 16S rRNA sequences was used (Fig. 1). In Figure 1, accession numbers in NCBI GenBank are given for each strain with its species name. Several dozens of species belong to Rhodococcus. To make the picture of the tree clearer, only the species that are close to R. qingshengii VKM Ac-2784D are shown. Two closest species are R. qingshengii and R. erythropolis. Both of them are well-known degraders of oil components (Táncsics et al., 2015).

The selection of related strains with oil-degradation activity

**Fig. 2.**
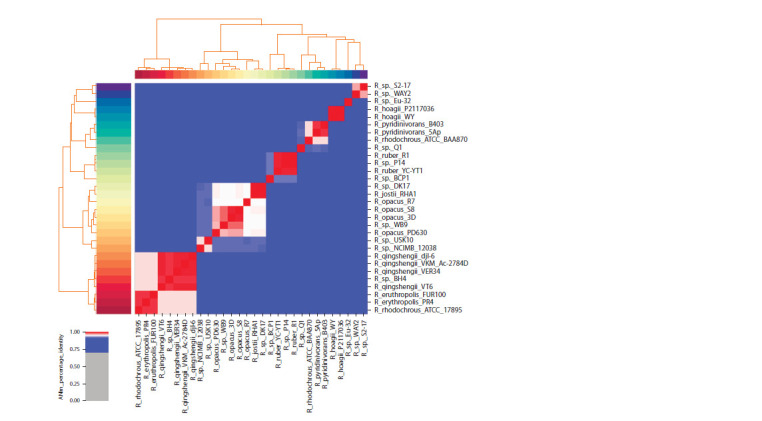
Heatmap and the phylogenetic tree of R. qingshengii VKM Ac-2784D and related species possessing oil-degrading activity Scale ANIm_percentage_identity shows the similarity percentage of a given pair of the genomes (from 0 to 1).

Although more than 50 rhodococci are known, we focused our attention on the species capable of degrading oil components, and the whole genome of which was disclosed. After literature analysis, 30 Rhodococcus genomes were selected for further study. For these species, the whole-genome and phylogenetic tree were built on the basis of average nucleotide identity distance matrix (presented as heatmap) (Fig. 2). This approach permits to consider the whole genomic data and not only differences in 16S rRNA fragments. Despite the fact that species of some strains were not determined, it was suggested that Rhodococcus sp. BH4, like R. qingshengii VKM Ac- 2784D, is referred to R. qingshengii. It should be noted that R. erythropolis and R. rhodochrous ATCC 17895 species differ considerably from R. qingshengii. Interestingly enough, one of the first known oil-degrading strains R. jostii RHA1 is very close to Rhodococcus sp. DK17.

Gene clusters of alkanes degradation

**Fig. 3.**
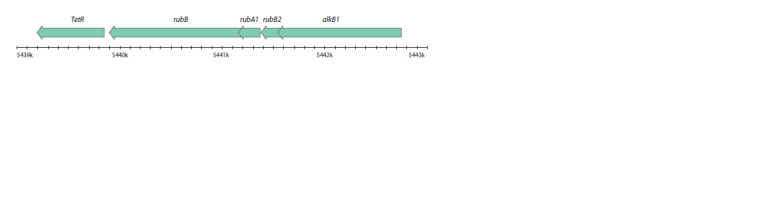
The structure of gene cluster encoding alkane-monooxigenase, rubredoxins and rubredoxin reductase in the R. qingshengii VKM Ac-2784D chromosome Here and in Figures 4 and 5: arrows show transcription direction.

It was previously reported that the core gene of alkane degradation, alkB, is usually co-located with genes of rubredoxin (alkG1, alkG2 or rubA1, rubA2) or rubredoxin reductase (alkT or rubB) (Whyte et al., 2002). In the present work, it was shown that R. qingshengii VKM Ac-2784D genome has two clusters containing genes of alkane-monooxigenase and rubredoxins (Fig. 3). Also, five separately located genes of alkane-monooxigenase were found (three in chromosome and two in plasmid). The R. qingshengii VKM Ac-2784D genome includes 14 genes of cytochrome P450 (11 in chromosome and 3 in plasmid) and has no SDIMO encoding genes.

Central pathways of PAHs destruction

**Fig. 4.**
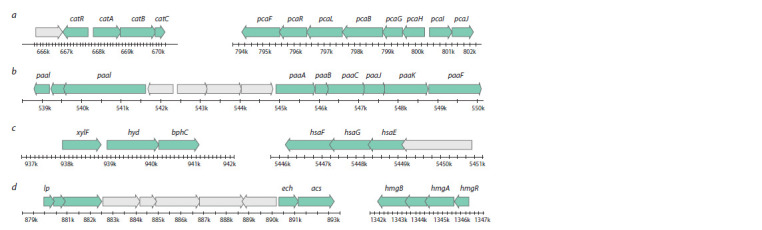
Gene clusters structure of four central pathways in the R. qingshengii VKM Ac-2784D genome: β-ketoadipate (a), phenylacetate (b), 2-hydroxypentadienoate (c), homogentisate (d ).

Four central pathways of destruction were revealed for R. qingshengii VKM Ac-2784D: β-ketoadipate, phenylacetate, 2-hydroxypentadienoate, homogentisate. The structure of the gene clusters (Fig. 4) is similar to that of the previously described strains: R. jostii RHA1, R. ruber Chol-4 (Navarro- Llorens et al., 2005; Yam et al., 2010; Gibu et al., 2019; Guevara et al., 2019).

Peripheral pathways of PAHs destruction

Generally, the aromatic ring is cleaved by dioxygenase systems Rieske 2Fe-2S, which are involved in the degradation of biphenyls, ethylbenzene and naphthalene (gene clusters including the bph, etb and nah genes). These systems possess a wide range of substrate specificity and can be present in the Rhodococcus genome simultaneously (Shumkova et al., 2015). The R. qingshengii VKM Ac-2784D genome contains 88 probable oxygenases: 25 dioxygenases and 63 monooxygenases.

**Fig. 5.**
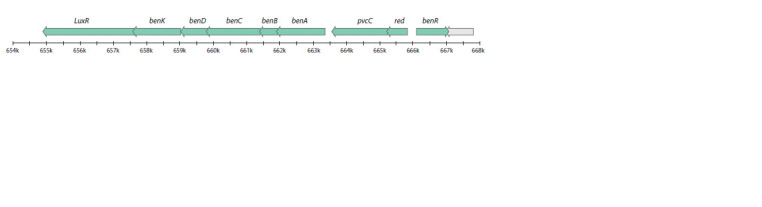
Structure of gene clusters encoding peripheral pathways of PAHs degradation in the R. qingshengii VKM Ac-2784D genome: benzoic acid degradation.

Several gene clusters in the R. qingshengii VKM Ac-2784D genome belong to peripheral pathways. Among them are 1,2-dioxygenase, 2,3-dioxygenase, benzoic acid degradation gene as well as two genes of biphenyl-2,3-diol, which participate in biphenyl catabolism. The structure of the bphABCDK gene cluster is shown in Figure 5. Moreover, the genes encoding dioxygenases, responsible for the intradiol and extradiol aromatic ring-cleavage, were found. At the same time, the R. qingshengii VKM Ac-2784D genome contains no tmo gene cluster, which takes part in transformation of toluene to n-cresol.

Biosurfactants synthesis

For some Rhodococcus species, gene clusters related to the trehalose-based synthesis of biosurfactants were documented. The pioneering works dedicated to synthetic approaches to such surfactants dealt with the pathogenic bacteria Mycobacterium tuberculosis (De Smet et al., 2000). Similar approaches were proposed for Rhodococcus species (Retamal-Morales et al., 2018). The R. qingshengii VKM Ac-2784D genome contains genes encoding proteins of the biosurfactants synthesis: otsA, otsB, treY, treZ.

## Discussion

The ability of the R. qingshengii VKM Ac-2784D strain to degrade oil components, PAHs and some other pollutants is supported by the results of our previous experimental studies (Tretyakova et al., 2019a, b; Belovezhets et al., 2020). It was shown that this strain mainly degraded the alkane fraction of oil, in particular С14–С24 alkanes (Belovezhets et al., 2021a). In addition, PAHs turned out to be also efficient in experiments with degradation of model oil compounds (Belovezhets et al., 2021b). Such metabolic activity can be explained by the synthesis of biosurfactants. The strain under study appeared to be an effective producer of extracellular and cell-binded forms of bio-SAAs. The highest amount of the synthesized extracellular biosurfactants was almost 1.5 g/L (substrate optimization was not carried out) (Belovezhets et al., 2021b).

To determine the mechanisms of PAH and alkane degradation, the whole genome sequencing and analysis of the gene clusters related to the corresponding metabolic pathways were performed.

Nowadays genome sequencing has become a routine procedure for studying gene properties. Gene annotation of a major part of genes (related to catabolic potential) is performed automatically with NCBI GenBank Prokaryotic Genome Annotation Pipeline (PGAP). These annotations allow some metabolic gene clusters to be determined at the stage of preliminary bioinformatic analysis. Further, BLAST search was employed to find particular functional genes from previous studies (Navarro-Llorens et al., 2005; Yam et al., 2010; Guevara et al., 2019). It was revealed that the R. qingshengii VKM Ac-2784D genome contains gene clusters 4 out of 8 known central pathways for PAHs destruction. Alkanes degradation enzymes are represented by two gene clusters containing genes of alkane-monooxigenase and rubredoxins and five separately located alkB genes (three in chromosome and two in plasmid). The presence of biphenyl 2,3-dioxygenese genes as well as gene clusters of benzoate and 2-hydroxypentandienoate indicates that R. qingshengii VKM Ac-2784D can degrade polychlorinated biphenyls

The detailed data on the studied genes and their presence in genomes of the strains are given in Supplementary Material1. For the R. qingshengii VKM Ac-2784D genome, the loci names of functional genes, references to the experimental works and degree of similarity for each gene are indicated. It is found that 7 out of 29 strains are most similar to R. qingshengii VKM Ac-2784D. These are R. ruber YC-YT1, R. qingshengii VER34, R. qingshengii VT6, R. qingshengii djl-6-2, Rhodococcus sp. PR4, Rhodococcus sp. BH4, R. rhodochrous ATCC 17895. The names of these strains are marked in gray in Supplementary Material.

Supplementary Materials are available in the online version of the paper: https://vavilovj-icg.ru/download/pict-2023-27/appx10.pdf


The bioinformatics data are supported by the results of previous biochemical studies. As a result, it is shown that the catabolic properties of R. qingshengii VKM Ac-2784D permit to apply this strain for the destruction of various pollutants.

## Conclusion

Nowadays there many genes and pathways related to the degradation of the various pollutants are identified. Genome analysis allows to uncover the metabolic potential of the particular microorganism, and obtain the specialized microbial mixtures with wide bioactive degradation spectrum. To understand the catabolic potential of these bacteria to degrade oil and its components we study its gene clusters, associated with such abilities. The structure of gene clusters is the same of known for strains R. jostii RHA1 and R. ruber Chol-4. Alkanes destruction genes grouped by two clusters and five separate genes alkB. The biodegradation activity may be enhanced by the biosurfactants, which are known to be synthesized by Rhodococcus. This knowledge can help to create the mixture of species with wide variation of metabolic pathways, but to evaluate the effectiveness of such mixtures further experiments are required.

## Conflict of interest

The authors declare no conflict of interest.
